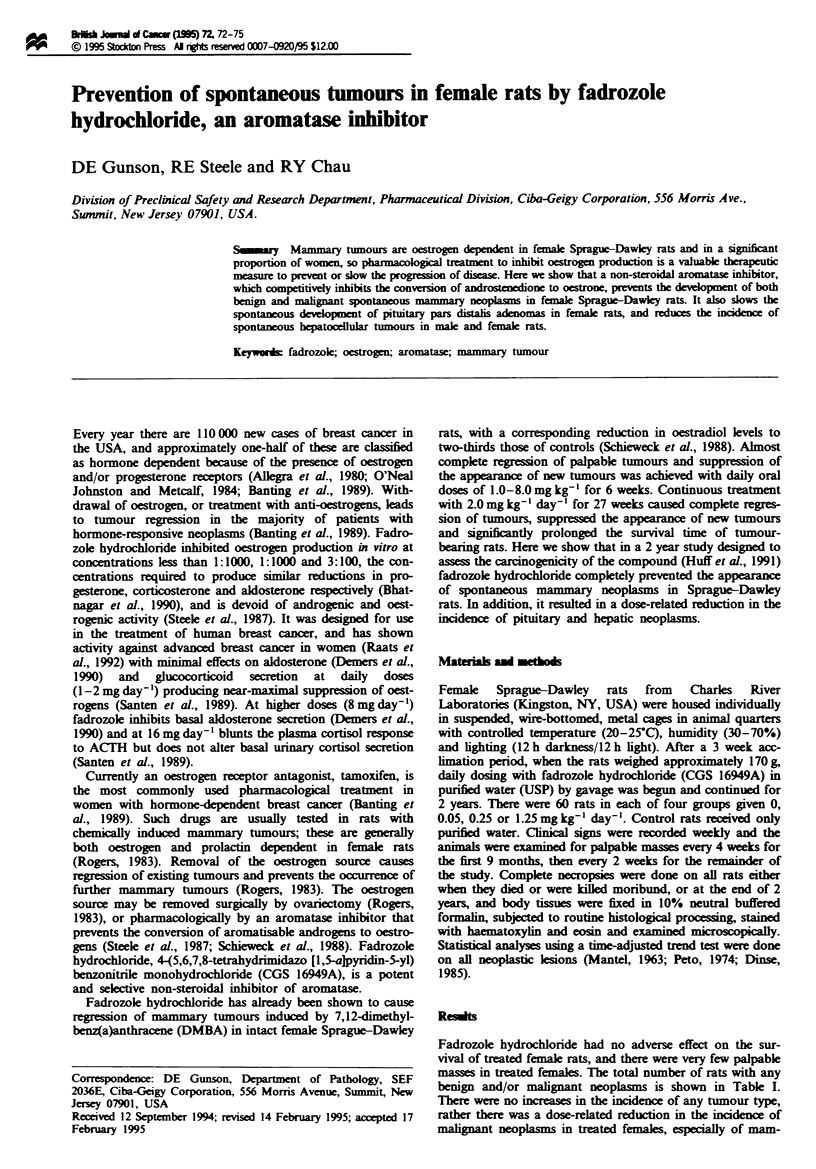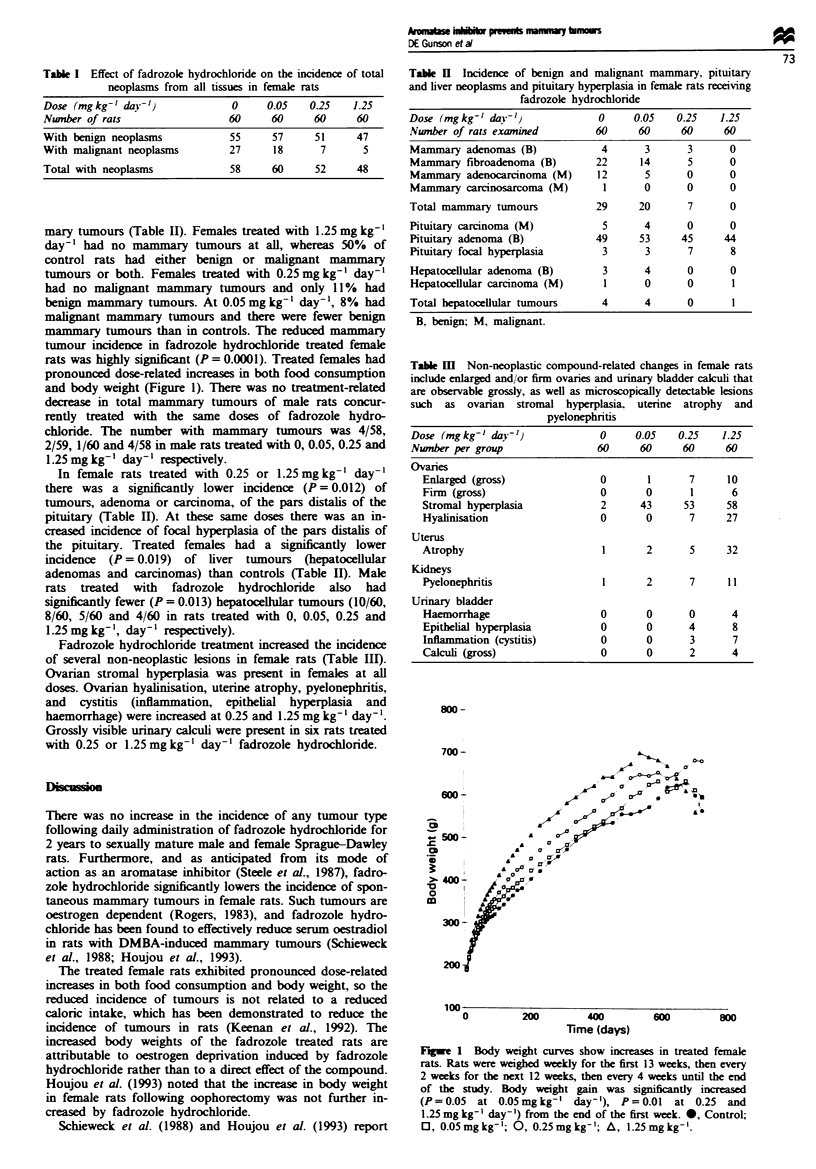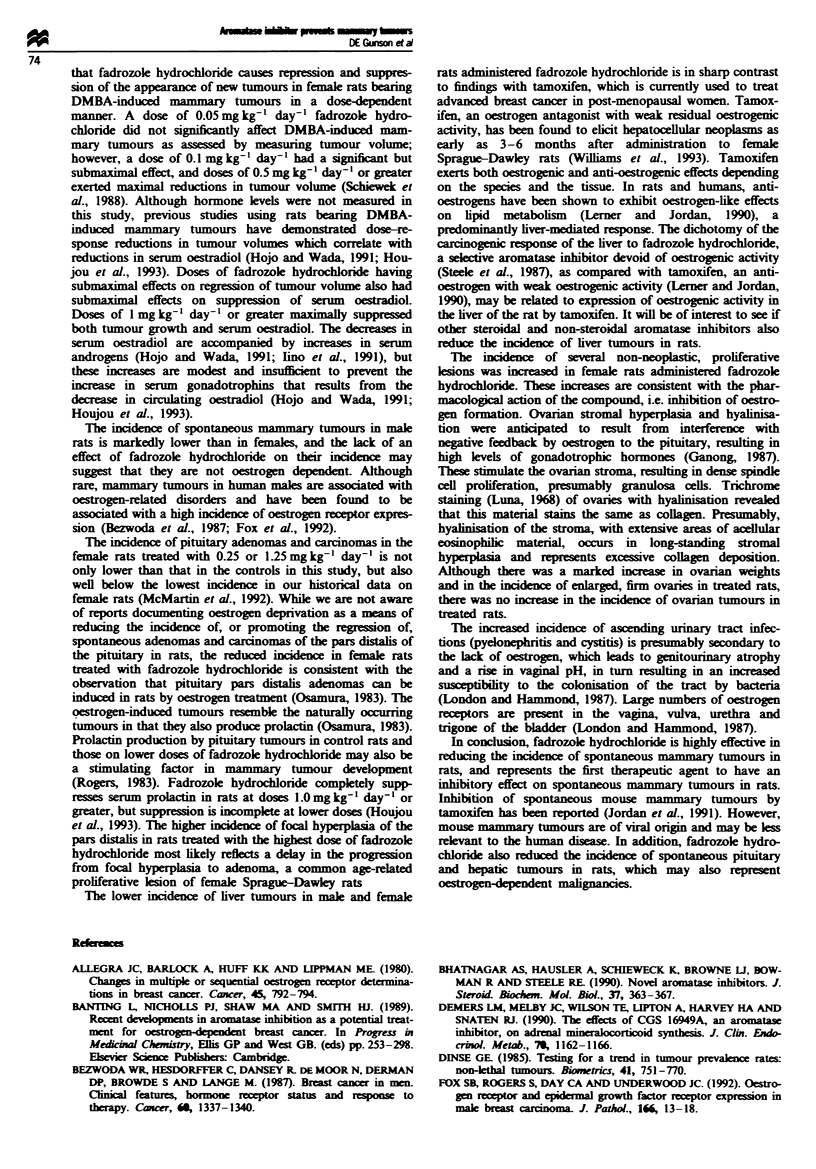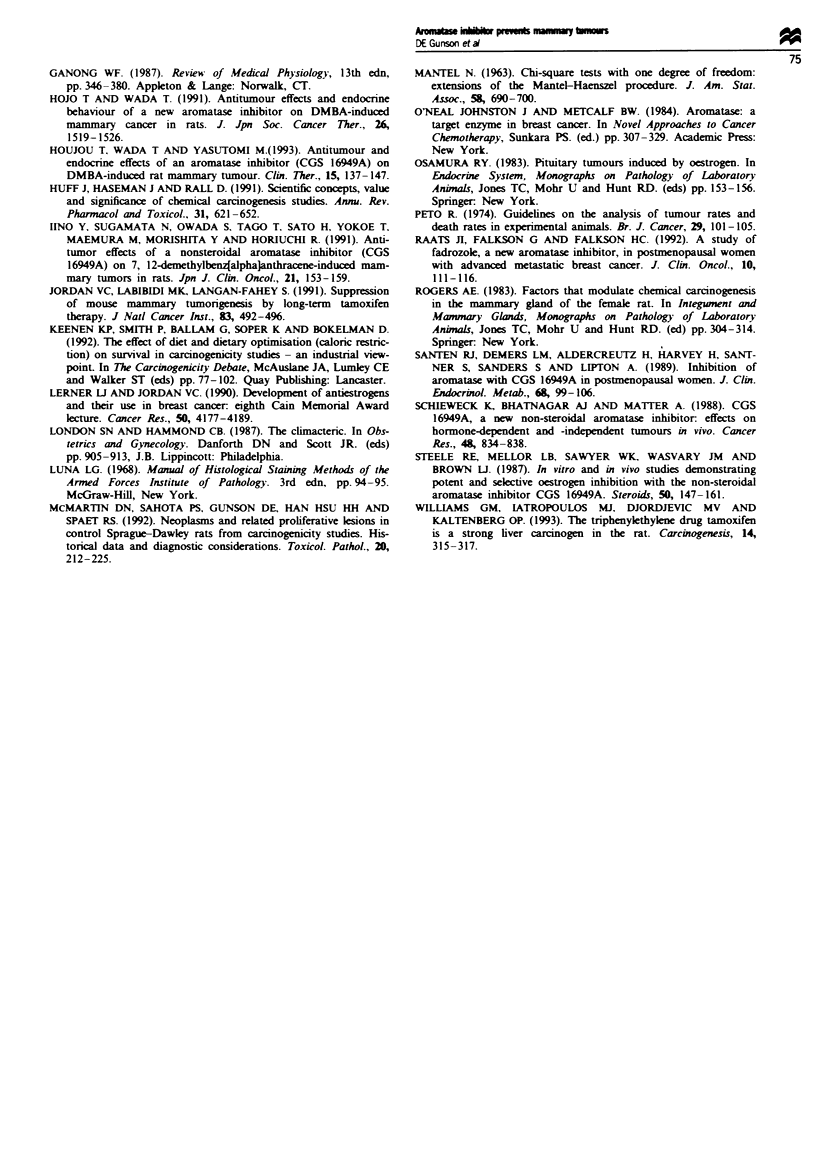# Prevention of spontaneous tumours in female rats by fadrozole hydrochloride, an aromatase inhibitor.

**DOI:** 10.1038/bjc.1995.279

**Published:** 1995-07

**Authors:** D. E. Gunson, R. E. Steele, R. Y. Chau

**Affiliations:** Division of Preclinical Safety, Ciba-Geigy Corporation, Summit, New Jersey 07901, USA.

## Abstract

Mammary tumours are oestrogen dependent in female Sprague-Dawley rats and in a significant proportion of women, so pharmacological treatment to inhibit oestrogen production is a valuable therapeutic measure to prevent or slow the progression of disease. Here we show that a non-steroidal aromatase inhibitor, which competitively inhibits the conversion of androstenedione to oestrone, prevents the development of both benign and malignant spontaneous mammary neoplasms in female Sprague-Dawley ats. It also slows the spontaneous development of pituitary pars distalis adenomas in female rats, and reduces the incidence of spontaneous hepatocellular tumours in male and female rats.


© 1995 Stockton Press All rights reserved 0007-0920/95 $12.00

### Prevention of spontaneous tumours in female rats by fadrozole hydrochloride, an aromatase inhibitor

DE Gunson, RE Steele and RY Chau

*Division of Preclinical Safety and Research Department, Pharmaceutical Division, Ciba-Geigy Corporation, 556 Morris Ave., Summit, New Jersey 07901, USA.*

**Summary** Mammary tumours are oestrogen dependent in female Sprague-Dawley rats and in a significant proportion of women, so pharmacological treatment to inhibit oestrogen production is a valuable therapeutic measure to prevent or slow the progression of disease. Here we show that a non-steroidal aromatase inhibitor, which competitively inhibits the conversion of androstenedione to oestrone, prevents the development of both benign and malignant spontaneous mammary neoplasms in female Sprague-Dawley rats. It also slows the spontaneous development of pituitary pars distalis adenomas in female rats, and reduces the incidence of spontaneous hepatocellular tumours in male and female rats.

**Keywords:** fadrozole; oestrogen; aromatase; mammary tumour

Every year there are 110 000 new cases of breast cancer in the USA, and approximately one-half of these are classified as hormone dependent because of the presence of oestrogen and/or progesterone receptors (Allegra *et al.*, 1980; O'Neal Johnston and Metcalf, 1984; Banting *et al.*, 1989). Withdrawal of oestrogen, or treatment with anti-oestrogens, leads to tumour regression in the majority of patients with hormone-responsive neoplasms (Banting *et al.*, 1989). Fadrozole hydrochloride inhibited oestrogen production *in vitro* at concentrations less than 1:1000, 1:1000 and 3:100, the concentrations required to produce similar reductions in progesterone, corticosterone and aldosterone respectively (Bhatnagar *et al.*, 1990), and is devoid of androgenic and oestrogenic activity (Steele *et al.*, 1987). It was designed for use in the treatment of human breast cancer, and has shown activity against advanced breast cancer in women (Raats *et al.*, 1992) with minimal effects on aldosterone (Demers *et al.*, 1990) and glucocorticoid secretion at daily doses ($1-2$ mg day$^{-1}$) producing near-maximal suppression of oestrogens (Santen *et al.*, 1989). At higher doses (8 mg day$^{-1}$) fadrozole inhibits basal aldosterone secretion (Demers *et al.*, 1990) and at 16 mg day$^{-1}$ blunts the plasma cortisol response to ACTH but does not alter basal urinary cortisol secretion (Santen *et al.*, 1989).

Currently an oestrogen receptor antagonist, tamoxifen, is the most commonly used pharmacological treatment in women with hormone-dependent breast cancer (Banting *et al.*, 1989). Such drugs are usually tested in rats with chemically induced mammary tumours; these are generally both oestrogen and prolactin dependent in female rats (Rogers, 1983). Removal of the oestrogen source causes regression of existing tumours and prevents the occurrence of further mammary tumours (Rogers, 1983). The oestrogen source may be removed surgically by ovariectomy (Rogers, 1983), or pharmacologically by an aromatase inhibitor that prevents the conversion of aromatisable androgens to oestrogens (Steele *et al.*, 1987; Schieweck *et al.*, 1988). Fadrozole hydrochloride, 4-(5,6,7,8-tetrahydrimidazo [1,5-*a*]pyridin-5-yl) benzonitrile monohydrochloride (CGS 16949A), is a potent and selective non-steroidal inhibitor of aromatase.

Fadrozole hydrochloride has already been shown to cause regression of mammary tumours induced by 7,12-dimethylbenz(a)anthracene (DMBA) in intact female Sprague-Dawley rats, with a corresponding reduction in oestradiol levels to two-thirds those of controls (Schieweck *et al.*, 1988). Almost complete regression of palpable tumours and suppression of the appearance of new tumours was achieved with daily oral doses of 1.0-8.0 mg kg$^{-1}$ for 6 weeks. Continuous treatment with 2.0 mg kg$^{-1}$ day$^{-1}$ for 27 weeks caused complete regression of tumours, suppressed the appearance of new tumours and significantly prolonged the survival time of tumour-bearing rats. Here we show that in a 2 year study designed to assess the carcinogenicity of the compound (Huff *et al.*, 1991) fadrozole hydrochloride completely prevented the appearance of spontaneous mammary neoplasms in Sprague-Dawley rats. In addition, it resulted in a dose-related reduction in the incidence of pituitary and hepatic neoplasms.

### Materials and methods

Female Sprague-Dawley rats from Charles River Laboratories (Kingston, NY, USA) were housed individually in suspended, wire-bottomed, metal cages in animal quarters with controlled temperature (20-25°C), humidity (30-70%) and lighting (12 h darkness/12 h light). After a 3 week acclimation period, when the rats weighed approximately 170 g, daily dosing with fadrozole hydrochloride (CGS 16949A) in purified water (USP) by gavage was begun and continued for 2 years. There were 60 rats in each of four groups given 0, 0.05, 0.25 or 1.25 mg kg$^{-1}$ day$^{-1}$. Control rats received only purified water. Clinical signs were recorded weekly and the animals were examined for palpable masses every 4 weeks for the first 9 months, then every 2 weeks for the remainder of the study. Complete necropsies were done on all rats either when they died or were killed moribund, or at the end of 2 years, and body tissues were fixed in 10% neutral buffered formalin, subjected to routine histological processing, stained with haematoxylin and eosin and examined microscopically. Statistical analyses using a time-adjusted trend test were done on all neoplastic lesions (Mantel, 1963; Peto, 1974; Dinse, 1985).

### Results

Fadrozole hydrochloride had no adverse effect on the survival of treated female rats, and there were very few palpable masses in treated females. The total number of rats with any benign and/or malignant neoplasms is shown in Table I. There were no increases in the incidence of any tumour type, rather there was a dose-related reduction in the incidence of malignant neoplasms in treated females, especially of mam-

Correspondence: DE Gunson, Department of Pathology, SEF 2036E, Ciba-Geigy Corporation, 556 Morris Avenue, Summit, New Jersey 07901, USA
Received 12 September 1994; revised 14 February 1995; accepted 17 February 1995

**Table I** Effect of fadrozole hydrochloride on the incidence of total neoplasms from all tissues in female rats

| *Dose (mg kg⁻¹ day⁻¹)* | *0* | *0.05* | *0.25* | *1.25* |
|---|---|---|---|---|
| *Number of rats* | *60* | *60* | *60* | *60* |
| With benign neoplasms | 55 | 57 | 51 | 47 |
| With malignant neoplasms | 27 | 18 | 7 | 5 |
| Total with neoplasms | 58 | 60 | 52 | 48 |

mary tumours (Table II). Females treated with 1.25 mg $kg^{-1}$ $day^{-1}$ had no mammary tumours at all, whereas 50% of control rats had either benign or malignant mammary tumours or both. Females treated with 0.25 mg $kg^{-1}$ $day^{-1}$ had no malignant mammary tumours and only 11% had benign mammary tumours. At 0.05 mg $kg^{-1}$ $day^{-1}$, 8% had malignant mammary tumours and there were fewer benign mammary tumours than in controls. The reduced mammary tumour incidence in fadrozole hydrochloride treated female rats was highly significant ($P = 0.0001$). Treated females had pronounced dose-related increases in both food consumption and body weight (Figure 1). There was no treatment-related decrease in total mammary tumours of male rats concurrently treated with the same doses of fadrozole hydrochloride. The number with mammary tumours was 4/58, 2/59, 1/60 and 4/58 in male rats treated with 0, 0.05, 0.25 and 1.25 mg $kg^{-1}$ $day^{-1}$ respectively.

In female rats treated with 0.25 or 1.25 mg $kg^{-1}$ $day^{-1}$ there was a significantly lower incidence ($P = 0.012$) of tumours, adenoma or carcinoma, of the pars distalis of the pituitary (Table II). At these same doses there was an increased incidence of focal hyperplasia of the pars distalis of the pituitary. Treated females had a significantly lower incidence ($P = 0.019$) of liver tumours (hepatocellular adenomas and carcinomas) than controls (Table II). Male rats treated with fadrozole hydrochloride also had significantly fewer ($P = 0.013$) hepatocellular tumours (10/60, 8/60, 5/60 and 4/60 in rats treated with 0, 0.05, 0.25 and 1.25 mg $kg^{-1}$, $day^{-1}$ respectively).

Fadrozole hydrochloride treatment increased the incidence of several non-neoplastic lesions in female rats (Table III). Ovarian stromal hyperplasia was present in females at all doses. Ovarian hyalinisation, uterine atrophy, pyelonephritis, and cystitis (inflammation, epithelial hyperplasia and haemorrhage) were increased at 0.25 and 1.25 mg $kg^{-1}$ $day^{-1}$. Grossly visible urinary calculi were present in six rats treated with 0.25 or 1.25 mg $kg^{-1}$ $day^{-1}$ fadrozole hydrochloride.

### Discussion

There was no increase in the incidence of any tumour type following daily administration of fadrozole hydrochloride for 2 years to sexually mature male and female Sprague–Dawley rats. Furthermore, and as anticipated from its mode of action as an aromatase inhibitor (Steele *et al.*, 1987), fadrozole hydrochloride significantly lowers the incidence of spontaneous mammary tumours in female rats. Such tumours are oestrogen dependent (Rogers, 1983), and fadrozole hydrochloride has been found to effectively reduce serum oestradiol in rats with DMBA-induced mammary tumours (Schieweck *et al.*, 1988; Houjou *et al.*, 1993).

The treated female rats exhibited pronounced dose-related increases in both food consumption and body weight, so the reduced incidence of tumours is not related to a reduced caloric intake, which has been demonstrated to reduce the incidence of tumours in rats (Keenan *et al.*, 1992). The increased body weights of the fadrozole treated rats are attributable to oestrogen deprivation induced by fadrozole hydrochloride rather than to a direct effect of the compound. Houjou *et al.* (1993) noted that the increase in body weight in female rats following oophorectomy was not further increased by fadrozole hydrochloride.

Schieweck *et al.* (1988) and Houjou *et al.* (1993) report

**Table II** Incidence of benign and malignant mammary, pituitary and liver neoplasms and pituitary hyperplasia in female rats receiving fadrozole hydrochloride

| *Dose (mg kg⁻¹ day⁻¹)* | *0* | *0.05* | *0.25* | *1.25* |
|---|---|---|---|---|
| *Number of rats examined* | *60* | *60* | *60* | *60* |
| Mammary adenomas (B) | 4 | 3 | 3 | 0 |
| Mammary fibroadenoma (B) | 22 | 14 | 5 | 0 |
| Mammary adenocarcinoma (M) | 12 | 5 | 0 | 0 |
| Mammary carcinosarcoma (M) | 1 | 0 | 0 | 0 |
| Total mammary tumours | 29 | 20 | 7 | 0 |
| Pituitary carcinoma (M) | 5 | 4 | 0 | 0 |
| Pituitary adenoma (B) | 49 | 53 | 45 | 44 |
| Pituitary focal hyperplasia | 3 | 3 | 7 | 8 |
| Hepatocellular adenoma (B) | 3 | 4 | 0 | 0 |
| Hepatocellular carcinoma (M) | 1 | 0 | 0 | 1 |
| Total hepatocellular tumours | 4 | 4 | 0 | 1 |

B, benign; M, malignant.

**Table III** Non-neoplastic compound-related changes in female rats include enlarged and/or firm ovaries and urinary bladder calculi that are observable grossly, as well as microscopically detectable lesions such as ovarian stromal hyperplasia, uterine atrophy and pyelonephritis

| *Dose (mg kg⁻¹ day⁻¹)* | *0* | *0.05* | *0.25* | *1.25* |
|---|---|---|---|---|
| *Number per group* | *60* | *60* | *60* | *60* |
| Ovaries | | | | |
| Enlarged (gross) | 0 | 1 | 7 | 10 |
| Firm (gross) | 0 | 0 | 1 | 6 |
| Stromal hyperplasia | 2 | 43 | 53 | 58 |
| Hyalinisation | 0 | 0 | 7 | 27 |
| Uterus | | | | |
| Atrophy | 1 | 2 | 5 | 32 |
| Kidneys | | | | |
| Pyelonephritis | 1 | 2 | 7 | 11 |
| Urinary bladder | | | | |
| Haemorrhage | 0 | 0 | 0 | 4 |
| Epithelial hyperplasia | 0 | 0 | 4 | 8 |
| Inflammation (cystitis) | 0 | 0 | 3 | 7 |
| Calculi (gross) | 0 | 0 | 2 | 4 |

**Figure 1** Body weight curves show increases in treated female rats. Rats were weighed weekly for the first 13 weeks, then every 2 weeks for the next 12 weeks, then every 4 weeks until the end of the study. Body weight gain was significantly increased ($P = 0.05$ at 0.05 mg $kg^{-1}$ $day^{-1}$), $P = 0.01$ at 0.25 and 1.25 mg $kg^{-1}$ $day^{-1}$) from the end of the first week. ●, Control; □, 0.05 mg $kg^{-1}$; O, 0.25 mg $kg^{-1}$; Δ, 1.25 mg $kg^{-1}$.

that fadrozole hydrochloride causes repression and suppression of the appearance of new tumours in female rats bearing DMBA-induced mammary tumours in a dose-dependent manner. A dose of 0.05 mg $kg^{-1}$ $day^{-1}$ fadrozole hydrochloride did not significantly affect DMBA-induced mammary tumours as assessed by measuring tumour volume; however, a dose of 0.1 mg $kg^{-1}$ $day^{-1}$ had a significant but submaximal effect, and doses of 0.5 mg $kg^{-1}$ $day^{-1}$ or greater exerted maximal reductions in tumour volume (Schiewek *et al.*, 1988). Although hormone levels were not measured in this study, previous studies using rats bearing DMBA-induced mammary tumours have demonstrated dose–response reductions in tumour volumes which correlate with reductions in serum oestradiol (Hojo and Wada, 1991; Houjou *et al.*, 1993). Doses of fadrozole hydrochloride having submaximal effects on regression of tumour volume also had submaximal effects on suppression of serum oestradiol. Doses of 1 mg $kg^{-1}$ $day^{-1}$ or greater maximally suppressed both tumour growth and serum oestradiol. The decreases in serum oestradiol are accompanied by increases in serum androgens (Hojo and Wada, 1991; Iino *et al.*, 1991), but these increases are modest and insufficient to prevent the increase in serum gonadotrophins that results from the decrease in circulating oestradiol (Hojo and Wada, 1991; Houjou *et al.*, 1993).

The incidence of spontaneous mammary tumours in male rats is markedly lower than in females, and the lack of an effect of fadrozole hydrochloride on their incidence may suggest that they are not oestrogen dependent. Although rare, mammary tumours in human males are associated with oestrogen-related disorders and have been found to be associated with a high incidence of oestrogen receptor expression (Bezwoda *et al.*, 1987; Fox *et al.*, 1992).

The incidence of pituitary adenomas and carcinomas in the female rats treated with 0.25 or 1.25 mg $kg^{-1}$ $day^{-1}$ is not only lower than that in the controls in this study, but also well below the lowest incidence in our historical data on female rats (McMartin *et al.*, 1992). While we are not aware of reports documenting oestrogen deprivation as a means of reducing the incidence of, or promoting the regression of, spontaneous adenomas and carcinomas of the pars distalis of the pituitary in rats, the reduced incidence in female rats treated with fadrozole hydrochloride is consistent with the observation that pituitary pars distalis adenomas can be induced in rats by oestrogen treatment (Osamura, 1983). The oestrogen-induced tumours resemble the naturally occurring tumours in that they also produce prolactin (Osamura, 1983). Prolactin production by pituitary tumours in control rats and those on lower doses of fadrozole hydrochloride may also be a stimulating factor in mammary tumour development (Rogers, 1983). Fadrozole hydrochloride completely suppresses serum prolactin in rats at doses 1.0 mg $kg^{-1}$ $day^{-1}$ or greater, but suppression is incomplete at lower doses (Houjou *et al.*, 1993). The higher incidence of focal hyperplasia of the pars distalis in rats treated with the highest dose of fadrozole hydrochloride most likely reflects a delay in the progression from focal hyperplasia to adenoma, a common age-related proliferative lesion of female Sprague–Dawley rats

The lower incidence of liver tumours in male and female rats administered fadrozole hydrochloride is in sharp contrast to findings with tamoxifen, which is currently used to treat advanced breast cancer in post-menopausal women. Tamoxifen, an oestrogen antagonist with weak residual oestrogenic activity, has been found to elicit hepatocellular neoplasms as early as 3–6 months after administration to female Sprague–Dawley rats (Williams *et al.*, 1993). Tamoxifen exerts both oestrogenic and anti-oestrogenic effects depending on the species and the tissue. In rats and humans, anti-oestrogens have been shown to exhibit oestrogen-like effects on lipid metabolism (Lerner and Jordan, 1990), a predominantly liver-mediated response. The dichotomy of the carcinogenic response of the liver to fadrozole hydrochloride, a selective aromatase inhibitor devoid of oestrogenic activity (Steele *et al.*, 1987), as compared with tamoxifen, an anti-oestrogen with weak oestrogenic activity (Lerner and Jordan, 1990), may be related to expression of oestrogenic activity in the liver of the rat by tamoxifen. It will be of interest to see if other steroidal and non-steroidal aromatase inhibitors also reduce the incidence of liver tumours in rats.

The incidence of several non-neoplastic, proliferative lesions was increased in female rats administered fadrozole hydrochloride. These increases are consistent with the pharmacological action of the compound, i.e. inhibition of oestrogen formation. Ovarian stromal hyperplasia and hyalinisation were anticipated to result from interference with negative feedback by oestrogen to the pituitary, resulting in high levels of gonadotrophic hormones (Ganong, 1987). These stimulate the ovarian stroma, resulting in dense spindle cell proliferation, presumably granulosa cells. Trichrome staining (Luna, 1968) of ovaries with hyalinisation revealed that this material stains the same as collagen. Presumably, hyalinisation of the stroma, with extensive areas of acellular eosinophilic material, occurs in long-standing stromal hyperplasia and represents excessive collagen deposition. Although there was a marked increase in ovarian weights and in the incidence of enlarged, firm ovaries in treated rats, there was no increase in the incidence of ovarian tumours in treated rats.

The increased incidence of ascending urinary tract infections (pyelonephritis and cystitis) is presumably secondary to the lack of oestrogen, which leads to genitourinary atrophy and a rise in vaginal pH, in turn resulting in an increased susceptibility to the colonisation of the tract by bacteria (London and Hammond, 1987). Large numbers of oestrogen receptors are present in the vagina, vulva, urethra and trigone of the bladder (London and Hammond, 1987).

In conclusion, fadrozole hydrochloride is highly effective in reducing the incidence of spontaneous mammary tumours in rats, and represents the first therapeutic agent to have an inhibitory effect on spontaneous mammary tumours in rats. Inhibition of spontaneous mouse mammary tumours by tamoxifen has been reported (Jordan *et al.*, 1991). However, mouse mammary tumours are of viral origin and may be less relevant to the human disease. In addition, fadrozole hydrochloride also reduced the incidence of spontaneous pituitary and hepatic tumours in rats, which may also represent oestrogen-dependent malignancies.

### References

ALLEGRA JC, BARLOCK A, HUFF KK AND LIPPMAN ME. (1980). Changes in multiple or sequential oestrogen receptor determinations in breast cancer. *Cancer*, **45**, 792–794.

BANTING L, NICHOLLS PJ, SHAW MA AND SMITH HJ. (1989). Recent developments in aromatase inhibition as a potential treatment for oestrogen-dependent breast cancer. In *Progress in Medicinal Chemistry*, Ellis GP and West GB. (eds) pp. 253–298. Elsevier Science Publishers: Cambridge.

BEZWODA WR, HESDORFFER C, DANSEY R, DE MOOR N, DERMAN DP, BROWDE S AND LANGE M. (1987). Breast cancer in men. Clinical features, hormone receptor status and response to therapy. *Cancer*, **60**, 1337–1340.

BHATNAGAR AS, HAUSLER A, SCHIEWECK K, BROWNE LJ, BOWMAN R AND STEELE RE. (1990). Novel aromatase inhibitors. *J. Steroid. Biochem. Mol. Biol.*, **37**, 363–367.

DEMERS LM, MELBY JC, WILSON TE, LIPTON A, HARVEY HA AND SNATEN RJ. (1990). The effects of CGS 16949A, an aromatase inhibitor, on adrenal mineralocorticoid synthesis. *J. Clin. Endocrinol. Metab.*, **70**, 1162–1166.

DINSE GE. (1985). Testing for a trend in tumour prevalence rates: non-lethal tumours. *Biometrics*, **41**, 751–770.

FOX SB, ROGERS S, DAY CA AND UNDERWOOD JC. (1992). Oestrogen receptor and epidermal growth factor receptor expression in male breast carcinoma. *J. Pathol.*, **166**, 13–18.

GANONG WF. (1987). *Review of Medical Physiology*, 13th edn, pp. 346–380. Appleton & Lange: Norwalk, CT.

HOJO T AND WADA T. (1991). Antitumour effects and endocrine behaviour of a new aromatase inhibitor on DMBA-induced mammary cancer in rats. *J. Jpn Soc. Cancer Ther.*, **26**, 1519–1526.

HOUJOU T, WADA T AND YASUTOMI M.(1993). Antitumour and endocrine effects of an aromatase inhibitor (CGS 16949A) on DMBA-induced rat mammary tumour. *Clin. Ther.*, **15**, 137–147.

HUFF J, HASEMAN J AND RALL D. (1991). Scientific concepts, value and significance of chemical carcinogenesis studies. *Annu. Rev. Pharmacol and Toxicol.*, **31**, 621–652.

IINO Y, SUGAMATA N, OWADA S, TAGO T, SATO H, YOKOE T, MAEMURA M, MORISHITA Y AND HORIUCHI R. (1991). Antitumor effects of a nonsteroidal aromatase inhibitor (CGS 16949A) on 7, 12-demethylbenz[alpha]anthracene-induced mammary tumors in rats. *Jpn J. Clin. Oncol.*, **21**, 153–159.

JORDAN VC, LABIBIDI MK, LANGAN-FAHEY S. (1991). Suppression of mouse mammary tumorigenesis by long-term tamoxifen therapy. *J Natl Cancer Inst.*, **83**, 492–496.

KEENEN KP, SMITH P, BALLAM G, SOPER K AND BOKELMAN D. (1992). The effect of diet and dietary optimisation (caloric restriction) on survival in carcinogenicity studies – an industrial viewpoint. In *The Carcinogenicity Debate*, McAuslane JA, Lumley CE and Walker ST (eds) pp. 77–102. Quay Publishing: Lancaster.

LERNER LJ AND JORDAN VC. (1990). Development of antiestrogens and their use in breast cancer: eighth Cain Memorial Award lecture. *Cancer Res.*, **50**, 4177–4189.

LONDON SN AND HAMMOND CB. (1987). The climacteric. In *Obstetrics and Gynecology*. Danforth DN and Scott JR. (eds) pp. 905–913, J.B. Lippincott: Philadelphia.

LUNA LG. (1968). *Manual of Histological Staining Methods of the Armed Forces Institute of Pathology*. 3rd edn, pp. 94–95. McGraw-Hill, New York.

McMARTIN DN, SAHOTA PS, GUNSON DE, HAN HSU HH AND SPAET RS. (1992). Neoplasms and related proliferative lesions in control Sprague–Dawley rats from carcinogenicity studies. Historical data and diagnostic considerations. *Toxicol. Pathol.*, **20**, 212–225.

MANTEL N. (1963). Chi-square tests with one degree of freedom: extensions of the Mantel–Haenszel procedure. *J. Am. Stat. Assoc.*, **58**, 690–700.

O'NEAL JOHNSTON J AND METCALF BW. (1984). Aromatase: a target enzyme in breast cancer. In *Novel Approaches to Cancer Chemotherapy*, Sunkara PS. (ed.) pp. 307–329. Academic Press: New York.

OSAMURA RY. (1983). Pituitary tumours induced by oestrogen. In *Endocrine System, Monographs on Pathology of Laboratory Animals*, Jones TC, Mohr U and Hunt RD. (eds) pp. 153–156. Springer: New York.

PETO R. (1974). Guidelines on the analysis of tumour rates and death rates in experimental animals. *Br. J. Cancer*, **29**, 101–105.

RAATS JI, FALKSON G AND FALKSON HC. (1992). A study of fadrozole, a new aromatase inhibitor, in postmenopausal women with advanced metastatic breast cancer. *J. Clin. Oncol.*, **10**, 111–116.

ROGERS AE. (1983). Factors that modulate chemical carcinogenesis in the mammary gland of the female rat. In *Integument and Mammary Glands, Monographs on Pathology of Laboratory Animals*, Jones TC, Mohr U and Hunt RD. (ed) pp. 304–314. Springer: New York.

SANTEN RJ, DEMERS LM, ALDERCREUTZ H, HARVEY H, SANTNER S, SANDERS S AND LIPTON A. (1989). Inhibition of aromatase with CGS 16949A in postmenopausal women. *J. Clin. Endocrinol. Metab.*, **68**, 99–106.

SCHIEWECK K, BHATNAGAR AJ AND MATTER A. (1988). CGS 16949A, a new non-steroidal aromatase inhibitor: effects on hormone-dependent and -independent tumours *in vivo*. *Cancer Res.*, **48**, 834–838.

STEELE RE, MELLOR LB, SAWYER WK, WASVARY JM AND BROWN LJ. (1987). *In vitro* and *in vivo* studies demonstrating potent and selective oestrogen inhibition with the non-steroidal aromatase inhibitor CGS 16949A. *Steroids*, **50**, 147–161.

WILLIAMS GM, IATROPOULOS MJ, DJORDJEVIC MV AND KALTENBERG OP. (1993). The triphenylethylene drug tamoxifen is a strong liver carcinogen in the rat. *Carcinogenesis*, **14**, 315–317.